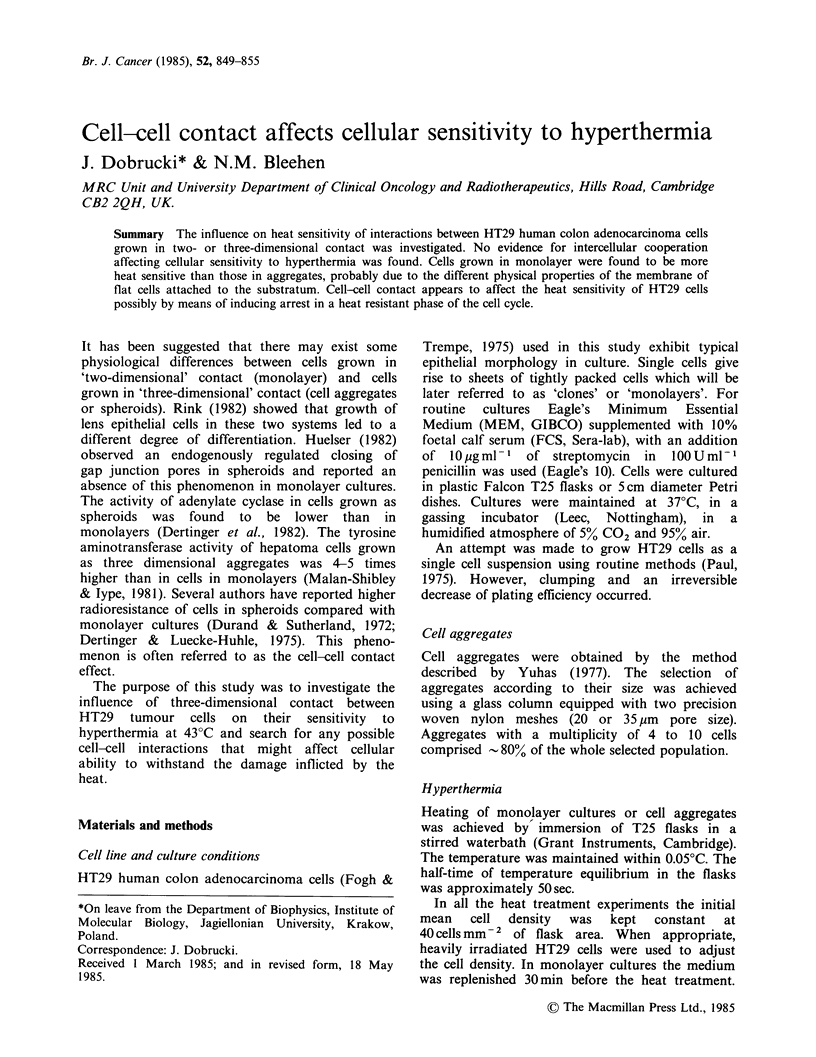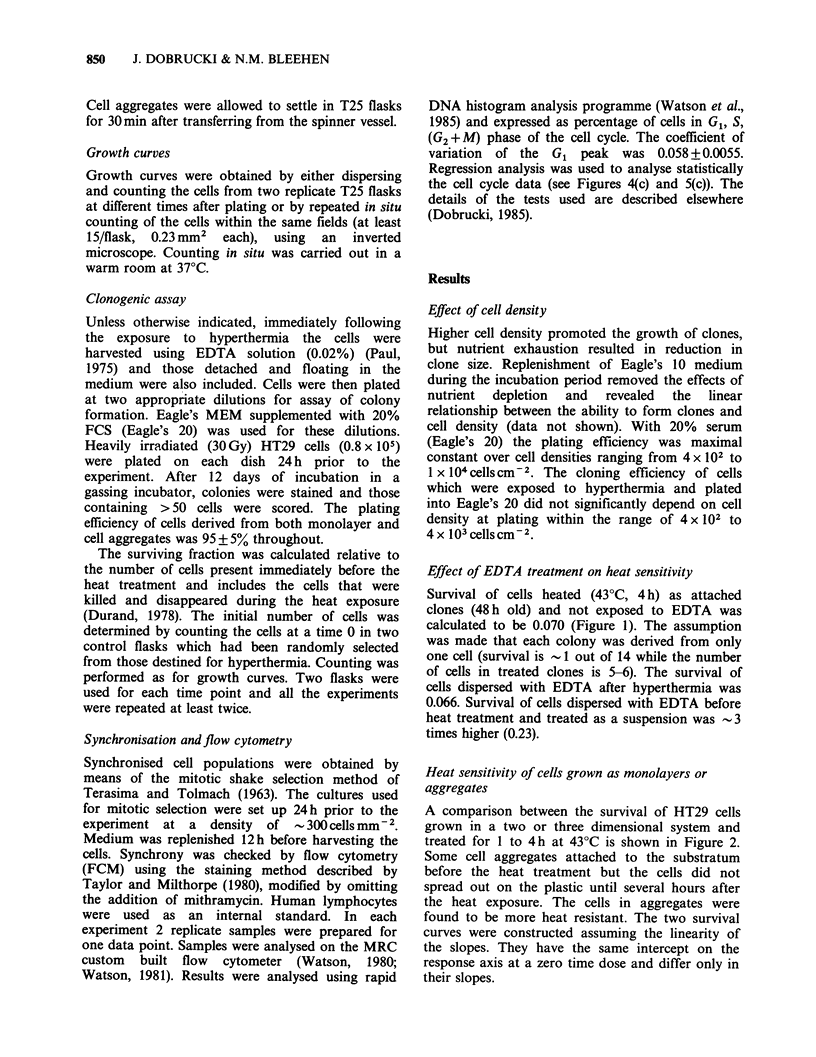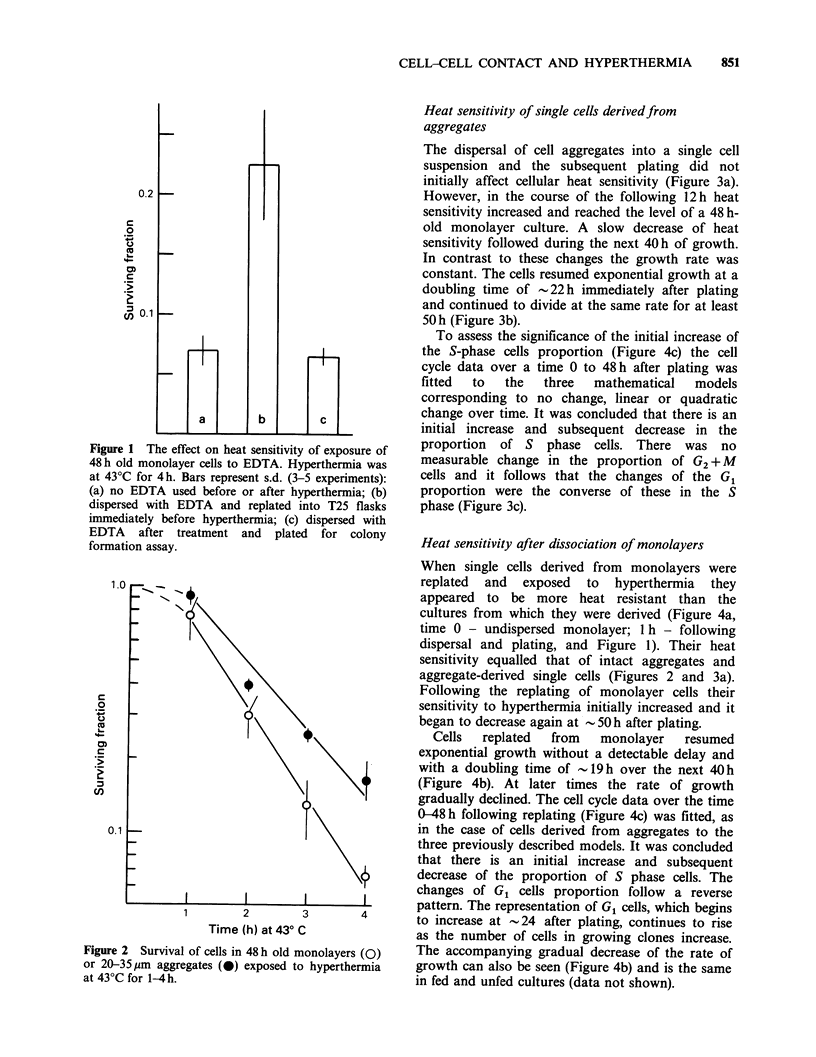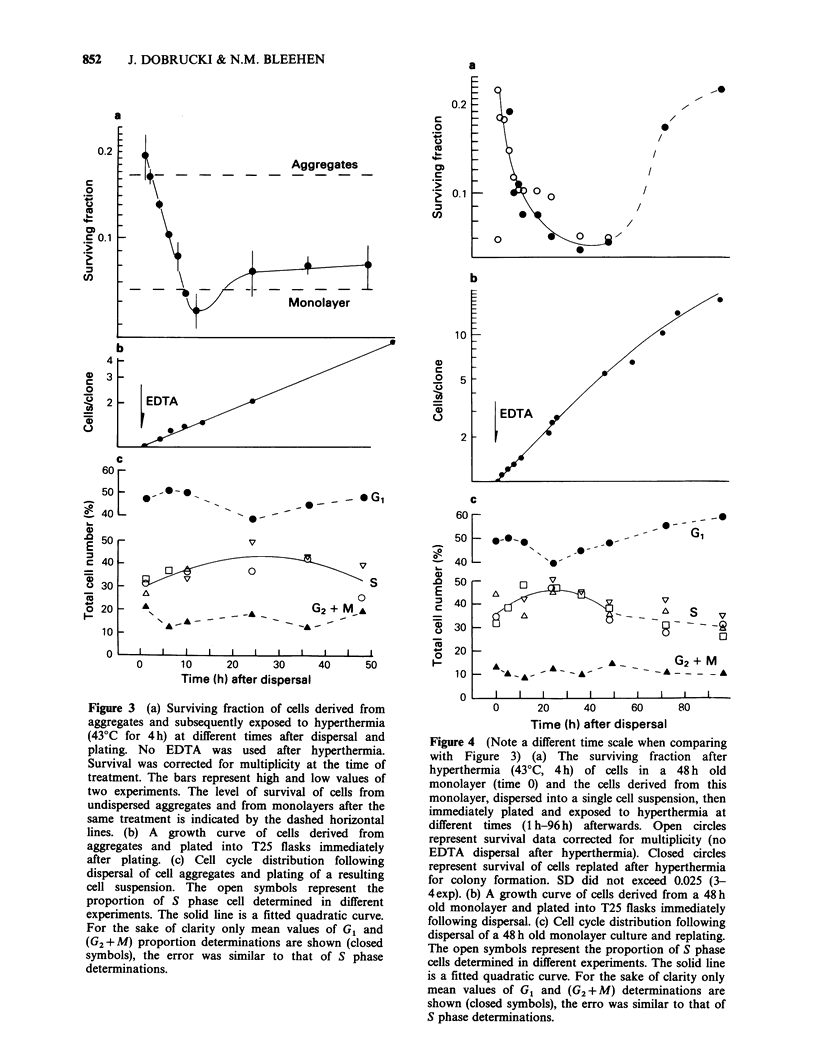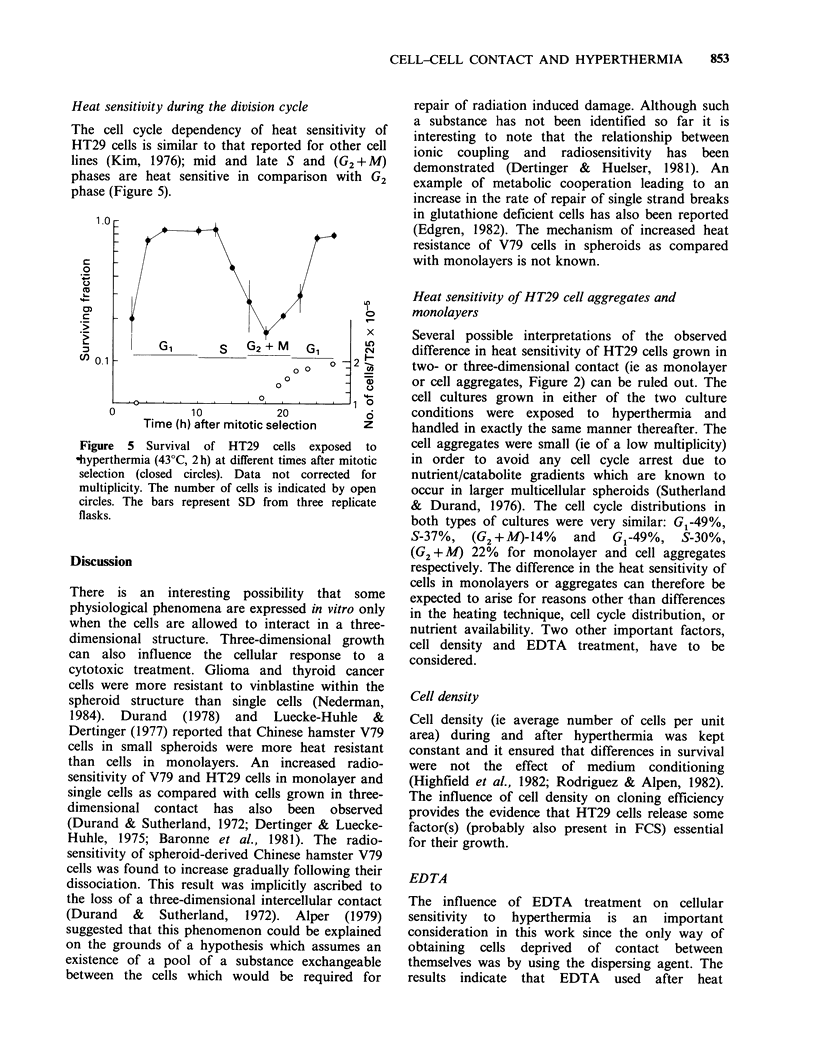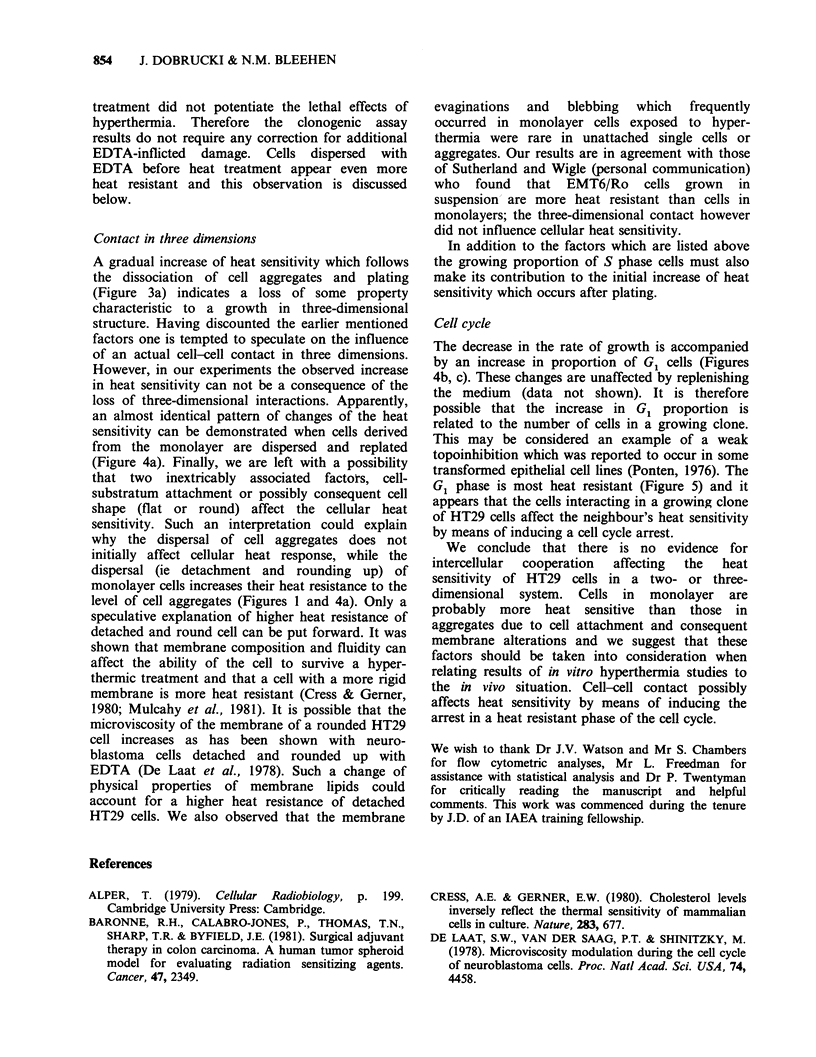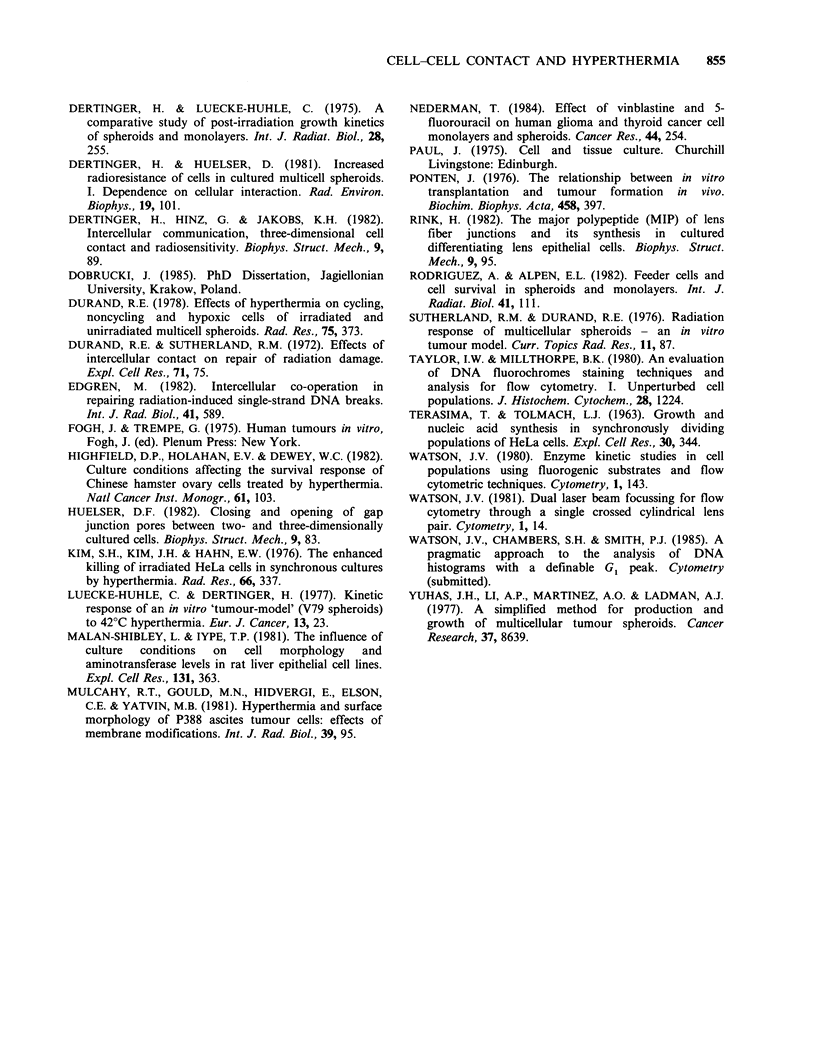# Cell-cell contact affects cellular sensitivity to hyperthermia.

**DOI:** 10.1038/bjc.1985.268

**Published:** 1985-12

**Authors:** J. Dobrucki, N. M. Bleehen

## Abstract

The influence on heat sensitivity of interactions between HT29 human colon adenocarcinoma cells grown in two- or three-dimensional contact was investigated. No evidence for intercellular cooperation affecting cellular sensitivity to hyperthermia was found. Cells grown in monolayer were found to be more heat sensitive than those in aggregates, probably due to the different physical properties of the membrane of flat cells attached to the substratum. Cell-cell contact appears to affect the heat sensitivity of HT29 cells possibly by means of inducing arrest in a heat resistant phase of the cell cycle.


*Br. J. Cancer* (1985), **52**, 849–855

### Cell–cell contact affects cellular sensitivity to hyperthermia

J. Dobrucki* & N.M. Bleehen

*MRC Unit and University Department of Clinical Oncology and Radiotherapeutics, Hills Road, Cambridge CB2 2QH, UK.*

**Summary** The influence on heat sensitivity of interactions between HT29 human colon adenocarcinoma cells grown in two- or three-dimensional contact was investigated. No evidence for intercellular cooperation affecting cellular sensitivity to hyperthermia was found. Cells grown in monolayer were found to be more heat sensitive than those in aggregates, probably due to the different physical properties of the membrane of flat cells attached to the substratum. Cell–cell contact appears to affect the heat sensitivity of HT29 cells possibly by means of inducing arrest in a heat resistant phase of the cell cycle.

It has been suggested that there may exist some physiological differences between cells grown in 'two-dimensional' contact (monolayer) and cells grown in 'three-dimensional' contact (cell aggregates or spheroids). Rink (1982) showed that growth of lens epithelial cells in these two systems led to a different degree of differentiation. Huelser (1982) observed an endogenously regulated closing of gap junction pores in spheroids and reported an absence of this phenomenon in monolayer cultures. The activity of adenylate cyclase in cells grown as spheroids was found to be lower than in monolayers (Dertinger *et al.*, 1982). The tyrosine aminotransferase activity of hepatoma cells grown as three dimensional aggregates was 4–5 times higher than in cells in monolayers (Malan-Shibley & Iype, 1981). Several authors have reported higher radioresistance of cells in spheroids compared with monolayer cultures (Durand & Sutherland, 1972; Dertinger & Luecke-Huhle, 1975). This phenomenon is often referred to as the cell–cell contact effect.

The purpose of this study was to investigate the influence of three-dimensional contact between HT29 tumour cells on their sensitivity to hyperthermia at 43°C and search for any possible cell–cell interactions that might affect cellular ability to withstand the damage inflicted by the heat.

### Materials and methods

### *Cell line and culture conditions*

HT29 human colon adenocarcinoma cells (Fogh & Trempe, 1975) used in this study exhibit typical epithelial morphology in culture. Single cells give rise to sheets of tightly packed cells which will be later referred to as 'clones' or 'monolayers'. For routine cultures Eagle's Minimum Essential Medium (MEM, GIBCO) supplemented with 10% foetal calf serum (FCS, Sera-lab), with an addition of $10\,\mu g\,ml^{-1}$ of streptomycin in $100\,U\,ml^{-1}$ penicillin was used (Eagle's 10). Cells were cultured in plastic Falcon T25 flasks or 5 cm diameter Petri dishes. Cultures were maintained at 37°C, in a gassing incubator (Leec, Nottingham), in a humidified atmosphere of 5% $CO_2$ and 95% air.

An attempt was made to grow HT29 cells as a single cell suspension using routine methods (Paul, 1975). However, clumping and an irreversible decrease of plating efficiency occurred.

### *Cell aggregates*

Cell aggregates were obtained by the method described by Yuhas (1977). The selection of aggregates according to their size was achieved using a glass column equipped with two precision woven nylon meshes (20 or 35 $\mu m$ pore size). Aggregates with a multiplicity of 4 to 10 cells comprised ~80% of the whole selected population.

### *Hyperthermia*

Heating of monolayer cultures or cell aggregates was achieved by immersion of T25 flasks in a stirred waterbath (Grant Instruments, Cambridge). The temperature was maintained within 0.05°C. The half-time of temperature equilibrium in the flasks was approximately 50 sec.

In all the heat treatment experiments the initial mean cell density was kept constant at $40\,cells\,mm^{-2}$ of flask area. When appropriate, heavily irradiated HT29 cells were used to adjust the cell density. In monolayer cultures the medium was replenished 30 min before the heat treatment.

*On leave from the Department of Biophysics, Institute of Molecular Biology, Jagiellonian University, Krakow, Poland.

Correspondence: J. Dobrucki.

Received 1 March 1985; and in revised form, 18 May 1985.

© The Macmillan Press Ltd., 1985

Cell aggregates were allowed to settle in T25 flasks for 30 min after transferring from the spinner vessel.

*Growth curves*

Growth curves were obtained by either dispersing and counting the cells from two replicate T25 flasks at different times after plating or by repeated *in situ* counting of the cells within the same fields (at least 15/flask, $0.23\,mm^2$ each), using an inverted microscope. Counting *in situ* was carried out in a warm room at 37°C.

*Clonogenic assay*

Unless otherwise indicated, immediately following the exposure to hyperthermia the cells were harvested using EDTA solution (0.02%) (Paul, 1975) and those detached and floating in the medium were also included. Cells were then plated at two appropriate dilutions for assay of colony formation. Eagle's MEM supplemented with 20% FCS (Eagle's 20) was used for these dilutions. Heavily irradiated (30 Gy) HT29 cells ($0.8 \times 10^5$) were plated on each dish 24 h prior to the experiment. After 12 days of incubation in a gassing incubator, colonies were stained and those containing >50 cells were scored. The plating efficiency of cells derived from both monolayer and cell aggregates was $95 \pm 5\%$ throughout.

The surviving fraction was calculated relative to the number of cells present immediately before the heat treatment and includes the cells that were killed and disappeared during the heat exposure (Durand, 1978). The initial number of cells was determined by counting the cells at a time 0 in two control flasks which had been randomly selected from those destined for hyperthermia. Counting was performed as for growth curves. Two flasks were used for each time point and all the experiments were repeated at least twice.

*Synchronisation and flow cytometry*

Synchronised cell populations were obtained by means of the mitotic shake selection method of Terasima and Tolmach (1963). The cultures used for mitotic selection were set up 24 h prior to the experiment at a density of $\sim 300\,cells\,mm^{-2}$. Medium was replenished 12 h before harvesting the cells. Synchrony was checked by flow cytometry (FCM) using the staining method described by Taylor and Milthorpe (1980), modified by omitting the addition of mithramycin. Human lymphocytes were used as an internal standard. In each experiment 2 replicate samples were prepared for one data point. Samples were analysed on the MRC custom built flow cytometer (Watson, 1980; Watson, 1981). Results were analysed using rapid DNA histogram analysis programme (Watson *et al.*, 1985) and expressed as percentage of cells in $G_1$, $S$, $(G_2+M)$ phase of the cell cycle. The coefficient of variation of the $G_1$ peak was $0.058 \pm 0.0055$. Regression analysis was used to analyse statistically the cell cycle data (see Figures 4(c) and 5(c)). The details of the tests used are described elsewhere (Dobrucki, 1985).

### Results

*Effect of cell density*

Higher cell density promoted the growth of clones, but nutrient exhaustion resulted in reduction in clone size. Replenishment of Eagle's 10 medium during the incubation period removed the effects of nutrient depletion and revealed the linear relationship between the ability to form clones and cell density (data not shown). With 20% serum (Eagle's 20) the plating efficiency was maximal constant over cell densities ranging from $4 \times 10^2$ to $1 \times 10^4\,cells\,cm^{-2}$. The cloning efficiency of cells which were exposed to hyperthermia and plated into Eagle's 20 did not significantly depend on cell density at plating within the range of $4 \times 10^2$ to $4 \times 10^3\,cells\,cm^{-2}$.

*Effect of EDTA treatment on heat sensitivity*

Survival of cells heated (43°C, 4 h) as attached clones (48 h old) and not exposed to EDTA was calculated to be 0.070 (Figure 1). The assumption was made that each colony was derived from only one cell (survival is ~1 out of 14 while the number of cells in treated clones is 5–6). The survival of cells dispersed with EDTA after hyperthermia was 0.066. Survival of cells dispersed with EDTA before heat treatment and treated as a suspension was ~3 times higher (0.23).

*Heat sensitivity of cells grown as monolayers or aggregates*

A comparison between the survival of HT29 cells grown in a two or three dimensional system and treated for 1 to 4 h at 43°C is shown in Figure 2. Some cell aggregates attached to the substratum before the heat treatment but the cells did not spread out on the plastic until several hours after the heat exposure. The cells in aggregates were found to be more heat resistant. The two survival curves were constructed assuming the linearity of the slopes. They have the same intercept on the response axis at a zero time dose and differ only in their slopes.

**Figure 1** The effect on heat sensitivity of exposure of 48 h old monolayer cells to EDTA. Hyperthermia was at 43°C for 4 h. Bars represent s.d. (3–5 experiments): (a) no EDTA used before or after hyperthermia; (b) dispersed with EDTA and replated into T25 flasks immediately before hyperthermia; (c) dispersed with EDTA after treatment and plated for colony formation assay.

**Figure 2** Survival of cells in 48 h old monolayers (○) or 20–35 μm aggregates (●) exposed to hyperthermia at 43°C for 1–4 h.

*Heat sensitivity of single cells derived from aggregates*

The dispersal of cell aggregates into a single cell suspension and the subsequent plating did not initially affect cellular heat sensitivity (Figure 3a). However, in the course of the following 12 h heat sensitivity increased and reached the level of a 48 h-old monolayer culture. A slow decrease of heat sensitivity followed during the next 40 h of growth. In contrast to these changes the growth rate was constant. The cells resumed exponential growth at a doubling time of ~22 h immediately after plating and continued to divide at the same rate for at least 50 h (Figure 3b).

To assess the significance of the initial increase of the *S*-phase cells proportion (Figure 4c) the cell cycle data over a time 0 to 48 h after plating was fitted to the three mathematical models corresponding to no change, linear or quadratic change over time. It was concluded that there is an initial increase and subsequent decrease in the proportion of *S* phase cells. There was no measurable change in the proportion of $G_2+M$ cells and it follows that the changes of the $G_1$ proportion were the converse of these in the *S* phase (Figure 3c).

*Heat sensitivity after dissociation of monolayers*

When single cells derived from monolayers were replated and exposed to hyperthermia they appeared to be more heat resistant than the cultures from which they were derived (Figure 4a, time 0 – undispersed monolayer; 1 h – following dispersal and plating, and Figure 1). Their heat sensitivity equalled that of intact aggregates and aggregate-derived single cells (Figures 2 and 3a). Following the replating of monolayer cells their sensitivity to hyperthermia initially increased and it began to decrease again at ~50 h after plating.

Cells replated from monolayer resumed exponential growth without a detectable delay and with a doubling time of ~19 h over the next 40 h (Figure 4b). At later times the rate of growth gradually declined. The cell cycle data over the time 0–48 h following replating (Figure 4c) was fitted, as in the case of cells derived from aggregates to the three previously described models. It was concluded that there is an initial increase and subsequent decrease of the proportion of *S* phase cells. The changes of $G_1$ cells proportion follow a reverse pattern. The representation of $G_1$ cells, which begins to increase at ~24 after plating, continues to rise as the number of cells in growing clones increase. The accompanying gradual decrease of the rate of growth can also be seen (Figure 4b) and is the same in fed and unfed cultures (data not shown).

**Figure 3** (a) Surviving fraction of cells derived from aggregates and subsequently exposed to hyperthermia (43°C for 4 h) at different times after dispersal and plating. No EDTA was used after hyperthermia. Survival was corrected for multiplicity at the time of treatment. The bars represent high and low values of two experiments. The level of survival of cells from undispersed aggregates and from monolayers after the same treatment is indicated by the dashed horizontal lines. (b) A growth curve of cells derived from aggregates and plated into T25 flasks immediately after plating. (c) Cell cycle distribution following dispersal of cell aggregates and plating of a resulting cell suspension. The open symbols represent the proportion of $S$ phase cell determined in different experiments. The solid line is a fitted quadratic curve. For the sake of clarity only mean values of $G_1$ and $(G_2+M)$ proportion determinations are shown (closed symbols), the error was similar to that of $S$ phase determinations.

**Figure 4** (Note a different time scale when comparing with Figure 3) (a) The surviving fraction after hyperthermia (43°C, 4 h) of cells in a 48 h old monolayer (time 0) and the cells derived from this monolayer, dispersed into a single cell suspension, then immediately plated and exposed to hyperthermia at different times (1 h–96 h) afterwards. Open circles represent survival data corrected for multiplicity (no EDTA dispersal after hyperthermia). Closed circles represent survival of cells replated after hyperthermia for colony formation. SD did not exceed 0.025 (3–4 exp). (b) A growth curve of cells derived from a 48 h old monolayer and plated into T25 flasks immediately following dispersal. (c) Cell cycle distribution following dispersal of a 48 h old monolayer culture and replating. The open symbols represent the proportion of $S$ phase cells determined in different experiments. The solid line is a fitted quadratic curve. For the sake of clarity only mean values of $G_1$ and $(G_2+M)$ determinations are shown (closed symbols), the erro was similar to that of $S$ phase determinations.

*Heat sensitivity during the division cycle*

The cell cycle dependency of heat sensitivity of HT29 cells is similar to that reported for other cell lines (Kim, 1976); mid and late *S* and $(G_2+M)$ phases are heat sensitive in comparison with $G_2$ phase (Figure 5).

**Figure 5** Survival of HT29 cells exposed to hyperthermia (43°C, 2 h) at different times after mitotic selection (closed circles). Data not corrected for multiplicity. The number of cells is indicated by open circles. The bars represent SD from three replicate flasks.

### Discussion

There is an interesting possibility that some physiological phenomena are expressed *in vitro* only when the cells are allowed to interact in a three-dimensional structure. Three-dimensional growth can also influence the cellular response to a cytotoxic treatment. Glioma and thyroid cancer cells were more resistant to vinblastine within the spheroid structure than single cells (Nederman, 1984). Durand (1978) and Luecke-Huhle & Dertinger (1977) reported that Chinese hamster V79 cells in small spheroids were more heat resistant than cells in monolayers. An increased radiosensitivity of V79 and HT29 cells in monolayer and single cells as compared with cells grown in three-dimensional contact has also been observed (Durand & Sutherland, 1972; Dertinger & Luecke-Huhle, 1975; Baronne *et al.*, 1981). The radiosensitivity of spheroid-derived Chinese hamster V79 cells was found to increase gradually following their dissociation. This result was implicitly ascribed to the loss of a three-dimensional intercellular contact (Durand & Sutherland, 1972). Alper (1979) suggested that this phenomenon could be explained on the grounds of a hypothesis which assumes an existence of a pool of a substance exchangeable between the cells which would be required for repair of radiation induced damage. Although such a substance has not been identified so far it is interesting to note that the relationship between ionic coupling and radiosensitivity has been demonstrated (Dertinger & Huelser, 1981). An example of metabolic cooperation leading to an increase in the rate of repair of single strand breaks in glutathione deficient cells has also been reported (Edgren, 1982). The mechanism of increased heat resistance of V79 cells in spheroids as compared with monolayers is not known.

*Heat sensitivity of HT29 cell aggregates and monolayers*

Several possible interpretations of the observed difference in heat sensitivity of HT29 cells grown in two- or three-dimensional contact (ie as monolayer or cell aggregates, Figure 2) can be ruled out. The cell cultures grown in either of the two culture conditions were exposed to hyperthermia and handled in exactly the same manner thereafter. The cell aggregates were small (ie of a low multiplicity) in order to avoid any cell cycle arrest due to nutrient/catabolite gradients which are known to occur in larger multicellular spheroids (Sutherland & Durand, 1976). The cell cycle distributions in both types of cultures were very similar: $G_1$-49%, *S*-37%, $(G_2+M)$-14% and $G_1$-49%, *S*-30%, $(G_2+M)$ 22% for monolayer and cell aggregates respectively. The difference in the heat sensitivity of cells in monolayers or aggregates can therefore be expected to arise for reasons other than differences in the heating technique, cell cycle distribution, or nutrient availability. Two other important factors, cell density and EDTA treatment, have to be considered.

*Cell density*

Cell density (ie average number of cells per unit area) during and after hyperthermia was kept constant and it ensured that differences in survival were not the effect of medium conditioning (Highfield *et al.*, 1982; Rodriguez & Alpen, 1982). The influence of cell density on cloning efficiency provides the evidence that HT29 cells release some factor(s) (probably also present in FCS) essential for their growth.

*EDTA*

The influence of EDTA treatment on cellular sensitivity to hyperthermia is an important consideration in this work since the only way of obtaining cells deprived of contact between themselves was by using the dispersing agent. The results indicate that EDTA used after heat treatment did not potentiate the lethal effects of hyperthermia. Therefore the clonogenic assay results do not require any correction for additional EDTA-inflicted damage. Cells dispersed with EDTA before heat treatment appear even more heat resistant and this observation is discussed below.

*Contact in three dimensions*

A gradual increase of heat sensitivity which follows the dissociation of cell aggregates and plating (Figure 3a) indicates a loss of some property characteristic to a growth in three-dimensional structure. Having discounted the earlier mentioned factors one is tempted to speculate on the influence of an actual cell–cell contact in three dimensions. However, in our experiments the observed increase in heat sensitivity can not be a consequence of the loss of three-dimensional interactions. Apparently, an almost identical pattern of changes of the heat sensitivity can be demonstrated when cells derived from the monolayer are dispersed and replated (Figure 4a). Finally, we are left with a possibility that two inextricably associated factors, cell-substratum attachment or possibly consequent cell shape (flat or round) affect the cellular heat sensitivity. Such an interpretation could explain why the dispersal of cell aggregates does not initially affect cellular heat response, while the dispersal (ie detachment and rounding up) of monolayer cells increases their heat resistance to the level of cell aggregates (Figures 1 and 4a). Only a speculative explanation of higher heat resistance of detached and round cell can be put forward. It was shown that membrane composition and fluidity can affect the ability of the cell to survive a hyperthermic treatment and that a cell with a more rigid membrane is more heat resistant (Cress & Gerner, 1980; Mulcahy *et al.*, 1981). It is possible that the microviscosity of the membrane of a rounded HT29 cell increases as has been shown with neuroblastoma cells detached and rounded up with EDTA (De Laat *et al.*, 1978). Such a change of physical properties of membrane lipids could account for a higher heat resistance of detached HT29 cells. We also observed that the membrane evaginations and blebbing which frequently occurred in monolayer cells exposed to hyperthermia were rare in unattached single cells or aggregates. Our results are in agreement with those of Sutherland and Wigle (personal communication) who found that EMT6/Ro cells grown in suspension are more heat resistant than cells in monolayers; the three-dimensional contact however did not influence cellular heat sensitivity.

In addition to the factors which are listed above the growing proportion of $S$ phase cells must also make its contribution to the initial increase of heat sensitivity which occurs after plating.

*Cell cycle*

The decrease in the rate of growth is accompanied by an increase in proportion of $G_1$ cells (Figures 4b, c). These changes are unaffected by replenishing the medium (data not shown). It is therefore possible that the increase in $G_1$ proportion is related to the number of cells in a growing clone. This may be considered an example of a weak topoinhibition which was reported to occur in some transformed epithelial cell lines (Ponten, 1976). The $G_1$ phase is most heat resistant (Figure 5) and it appears that the cells interacting in a growing clone of HT29 cells affect the neighbour's heat sensitivity by means of inducing a cell cycle arrest.

We conclude that there is no evidence for intercellular cooperation affecting the heat sensitivity of HT29 cells in a two- or three-dimensional system. Cells in monolayer are probably more heat sensitive than those in aggregates due to cell attachment and consequent membrane alterations and we suggest that these factors should be taken into consideration when relating results of *in vitro* hyperthermia studies to the *in vivo* situation. Cell–cell contact possibly affects heat sensitivity by means of inducing the arrest in a heat resistant phase of the cell cycle.

We wish to thank Dr J.V. Watson and Mr S. Chambers for flow cytometric analyses, Mr L. Freedman for assistance with statistical analysis and Dr P. Twentyman for critically reading the manuscript and helpful comments. This work was commenced during the tenure by J.D. of an IAEA training fellowship.

### References

ALPER, T. (1979). *Cellular Radiobiology*, p. 199. Cambridge University Press: Cambridge.

BARONNE, R.H., CALABRO-JONES, P., THOMAS, T.N., SHARP, T.R. & BYFIELD, J.E. (1981). Surgical adjuvant therapy in colon carcinoma. A human tumor spheroid model for evaluating radiation sensitizing agents. *Cancer*, **47**, 2349.

CRESS, A.E. & GERNER, E.W. (1980). Cholesterol levels inversely reflect the thermal sensitivity of mammalian cells in culture. *Nature*, **283**, 677.

DE LAAT, S.W., VAN DER SAAG, P.T. & SHINITZKY, M. (1978). Microviscosity modulation during the cell cycle of neuroblastoma cells. *Proc. Natl Acad. Sci. USA*, **74**, 4458.

DERTINGER, H. & LUECKE-HUHLE, C. (1975). A comparative study of post-irradiation growth kinetics of spheroids and monolayers. *Int. J. Radiat. Biol.*, **28**, 255.

DERTINGER, H. & HUELSER, D. (1981). Increased radioresistance of cells in cultured multicell spheroids. I. Dependence on cellular interaction. *Rad. Environ. Biophys.*, **19**, 101.

DERTINGER, H., HINZ, G. & JAKOBS, K.H. (1982). Intercellular communication, three-dimensional cell contact and radiosensitivity. *Biophys. Struct. Mech.*, **9**, 89.

DOBRUCKI, J. (1985). PhD Dissertation, Jagiellonian University, Krakow, Poland.

DURAND, R.E. (1978). Effects of hyperthermia on cycling, noncycling and hypoxic cells of irradiated and unirradiated multicell spheroids. *Rad. Res.*, **75**, 373.

DURAND, R.E. & SUTHERLAND, R.M. (1972). Effects of intercellular contact on repair of radiation damage. *Expl. Cell Res.*, **71**, 75.

EDGREN, M. (1982). Intercellular co-operation in repairing radiation-induced single-strand DNA breaks. *Int. J. Rad. Biol.*, **41**, 589.

FOGH, J. & TREMPE, G. (1975). Human tumours *in vitro*, Fogh, J. (ed). Plenum Press: New York.

HIGHFIELD, D.P., HOLAHAN, E.V. & DEWEY, W.C. (1982). Culture conditions affecting the survival response of Chinese hamster ovary cells treated by hyperthermia. *Natl Cancer Inst. Monogr.*, **61**, 103.

HUELSER, D.F. (1982). Closing and opening of gap junction pores between two- and three-dimensionally cultured cells. *Biophys. Struct. Mech.*, **9**, 83.

KIM, S.H., KIM, J.H. & HAHN, E.W. (1976). The enhanced killing of irradiated HeLa cells in synchronous cultures by hyperthermia. *Rad. Res.*, **66**, 337.

LUECKE-HUHLE, C. & DERTINGER, H. (1977). Kinetic response of an *in vitro* 'tumour-model' (V79 spheroids) to 42°C hyperthermia. *Eur. J. Cancer*, **13**, 23.

MALAN-SHIBLEY, L. & IYPE, T.P. (1981). The influence of culture conditions on cell morphology and aminotransferase levels in rat liver epithelial cell lines. *Expl. Cell Res.*, **131**, 363.

MULCAHY, R.T., GOULD, M.N., HIDVERGI, E., ELSON, C.E. & YATVIN, M.B. (1981). Hyperthermia and surface morphology of P388 ascites tumour cells: effects of membrane modifications. *Int. J. Rad. Biol.*, **39**, 95.

NEDERMAN, T. (1984). Effect of vinblastine and 5-fluorouracil on human glioma and thyroid cancer cell monolayers and spheroids. *Cancer Res.*, **44**, 254.

PAUL, J. (1975). Cell and tissue culture. Churchill Livingstone: Edinburgh.

PONTEN, J. (1976). The relationship between *in vitro* transplantation and tumour formation *in vivo*. *Biochim. Biophys. Acta*, **458**, 397.

RINK, H. (1982). The major polypeptide (MIP) of lens fiber junctions and its synthesis in cultured differentiating lens epithelial cells. *Biophys. Struct. Mech.*, **9**, 95.

RODRIGUEZ, A. & ALPEN, E.L. (1982). Feeder cells and cell survival in spheroids and monolayers. *Int. J. Radiat. Biol.* **41**, 111.

SUTHERLAND, R.M. & DURAND, R.E. (1976). Radiation response of multicellular spheroids – an *in vitro* tumour model. *Curr. Topics Rad. Res.*, **11**, 87.

TAYLOR, I.W. & MILLTHORPE, B.K. (1980). An evaluation of DNA fluorochromes staining techniques and analysis for flow cytometry. I. Unperturbed cell populations. *J. Histochem. Cytochem.*, **28**, 1224.

TERASIMA, T. & TOLMACH, L.J. (1963). Growth and nucleic acid synthesis in synchronously dividing populations of HeLa cells. *Expl. Cell Res.*, **30**, 344.

WATSON, J.V. (1980). Enzyme kinetic studies in cell populations using fluorogenic substrates and flow cytometric techniques. *Cytometry*, **1**, 143.

WATSON, J.V. (1981). Dual laser beam focussing for flow cytometry through a single crossed cylindrical lens pair. *Cytometry*, **1**, 14.

WATSON, J.V., CHAMBERS, S.H. & SMITH, P.J. (1985). A pragmatic approach to the analysis of DNA histograms with a definable $G_1$ peak. *Cytometry* (submitted).

YUHAS, J.H., LI, A.P., MARTINEZ, A.O. & LADMAN, A.J. (1977). A simplified method for production and growth of multicellular tumour spheroids. *Cancer Research*, **37**, 8639.